# c-Met enforces proinflammatory and migratory features of human activated CD4^+^ T cells

**DOI:** 10.1038/s41423-021-00721-9

**Published:** 2021-06-28

**Authors:** Mahdia Benkhoucha, Ngoc Lan Tran, Gautier Breville, Isis Senoner, Camilla Jandus, Patrice Lalive

**Affiliations:** 1 Department of Pathology and Immunology, Faculty of Medicine, University of Geneva, Geneva, Switzerland; 2 Department of Neurosciences, Division of Neurology, University Hospital of Geneva, Geneva, Switzerland; 3 Ludwig Institute for Cancer Research, Lausanne Branch, Lausanne, Switzerland

**Keywords:** Chronic inflammation, Immunology

The receptor tyrosine kinase c-Met is essential for embryonic development and tissue regeneration, as it promotes cell survival, proliferation, migration, and angiogenesis [1, 2]. The HGF/c-Met axis modulates several inflammatory-mediated diseases by acting on a wide variety of cells [3]. Nevertheless, studies on the role of c-Met in peripheral T cell functions are very limited, partly because we and others have reported negligible c-Met expression in naive T cells [2, 4]. Recently, we found that a fraction of murine cytotoxic CD8^+^ T lymphocytes (CTLs) expressed c-Met (c-Met^+^ CTLs). We also demonstrated the presence of c-Met^+^ CTLs in mouse tumors [5] and central nervous system (CNS) autoimmunity models [6]. Interestingly, c-Met^+^ CTL populations arise only under conditions caused by a pathological microenvironment. Hence, this particular c-Met^+^ population is barely detectable in tumor-free [5] and naïve (preimmunized) mice [7], suggesting that c-Met^+^ T cells are able to expand only after activation or in pathological settings. Based on these findings, we wondered whether c-Met expression can be induced on CD4^+^ T lymphocytes from human peripheral blood mononuclear cells (PBMCs) upon T cell receptor (TCR) triggering.

First, we assessed the expression of c-Met in circulating CD4^+^ T cells among freshly isolated PBMCs from healthy donors (HDs). Although Th17 and Th17.1 populations exhibited increased mRNA expression of c-Met, no or very weak c-Met protein levels were detected in the different CD4^+^ Th subsets as assessed by flow cytometry (Fig. S1A-D).

However, upon in vitro stimulation for 72 h with beads coated with anti-CD3/CD28 (to mimic TCR activation [8]), the mRNA and protein levels of c-Met expressed by CD4^+^ T cells increased significantly (Fig. 1A, Fig. S2A-B). Furthermore, we confirmed the increase in c-Met expression in differentiated CD4^+^ T cell subsets but not in naïve CD4^+^ T cells after anti-CD3/CD28 activation (Fig. 1B–D). Overall, these data suggest that mimicking TCR activation signals in vitro [9] is effective for the upregulation of c-Met on human CD4^+^ T lymphocytes.

**Fig. 1:**
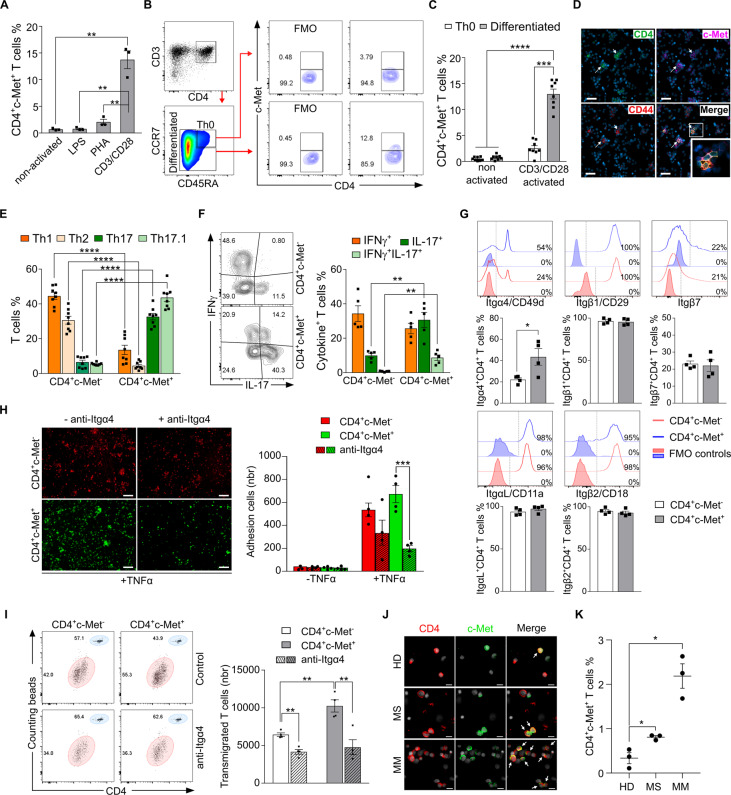
Human CD4^+^ T cells expressing c-Met display a proinflammatory and pro-migratory phenotype after TCR triggering. **A** PBMCs from HDs (*n* = 3) were stimulated 72 h with LPS, PHA, or anti-CD3/CD28-coated beads, and analyzed by flow cytometry for c-Met expression on CD4^+^ T cells (gated as described in Fig. S1A). **B** c-Met expression by CD4^+^ T cells differentiated or not (Th0) identified by flow cytometry. **C** c-Met^+^ quantification on Th0 cells (white bars) and differentiated CD4^+^ T cells (gray bars), non-activated and after anti-CD3/CD28 activation (*n* = 8 HDs). **D** Representative immunofluorescent images of PBMCs from HDs (*n* = 3) after anti-CD3/CD28 activation showing the expression of CD4 (green), c‐Met (pink), CD44 (red), and cell nuclei (blue) (scale bar = 50 μm). Triple positive cells are indicated with the arrows. Inset is 3× enlarged image. **E** Frequency of different Th populations (gated as described in Fig. S1A) were quantified by flow cytometry among the CD4^+^ T cells expressing or not c-Met (*n* = 8 HDs). **F** Representative flow cytometry contour plots and quantification of IFNγ and IL-17 expression by c-Met^−^ and c-Met^+^ CD4^+^ T cells after anti-CD3/CD28 activation (*n* = 5 HDs). **G** Representative flow cytometry histograms and quantification of Itgα4/CD49d, Itgβ1/CD29, Itgβ7, ItgαL/CD11a, and Itgβ2/CD18 expression on c-Met^−^ and c-Met^+^ CD4^+^ T cells after 72 h of activation with anti-CD3/CD28-coated beads (*n* = 4 HDs). **H** Adhesion assay of anti-CD3/CD28-activated c-Met^−^ and c-Met^+^ sorted CD4^+^ T cells treated or not with anti-Itgα4 antibody for 72 h, labeled with CellTrace^TM^ Far Red (red) or CFSE (green), respectively, and seeded on a monolayer of HUVEC cells (activated with TNFα). Quantification of adherent cells per well is shown (*n* = 4 HDs). Scale bar = 100 μm. **I** Transwell migration assay of anti-CD3/CD28-activated c-Met^−^ and c-Met^+^ sorted CD4^+^ T cells treated or not with anti-Itgα4 antibody for 72 h. The relative number of transmigrated cells was determined by flow cytometry using fluorescent counting beads (blue population) to normalize the number of transmigrated cells (red population). Quantification of the number of transmigrated cells is shown (*n* = 3 HDs). **J** Representative immunofluorescent images of cytospin PBMCs from HDs (*n* = 3), multiple sclerosis (MS) patients (*n* = 3), and malignant melanoma (MM) patients (*n* = 3), showing the expression of CD4 (red), c‐Met (green), and cell nuclei (white). Double positive cells are indicated with the arrows (scale bar = 20 μm). **K** Flow cytometry quantification of circulating CD4^+^c-Met^+^ T cells from HDs, MS, and MM PBMCs (*n* = 3). Data are presented as mean ± SEM; *P ≤ 0.05, **P ≤ 0.01, ***P ≤ 0.001, ****P ≤ 0.0001, unpaired two-tailed Student’s *t* test for two groups (**E, F, G, K**) or two-way ANOVA followed by Tukey’s post hoc test for multiple groups (**A, C, H, I**). The detailed methods are described in Supplementary information

To investigate the phenotypic properties of T cell subsets expressing c-Met, freshly isolated PBMCs from eight HDs were activated with anti-CD3/CD28-coated beads for 72 h. The expression of c-Met was predominantly observed in Th17 and Th17.1 subsets, whereas Th1 and Th2 subsets expressed low levels of c-Met (Fig. 1E, Fig. S2C). Next, we conducted flow cytometry analysis to assess the cytokine secretion profiles after anti-CD3/CD28 activation and PMA/ionomycin stimulation for 4 h prior to intracellular cytokine staining. The CD4^+^c-Met^+^ T cell subset produced a higher level of IL-17 both alone (Th17) and together with IFNγ (double positive, Th17.1) than did the CD4^+^c-Met^-^ subset (Fig. 1F). Taken together, these data show that Th17- and Th17.1-activated cells expressing c-Met are more prone to secreting the proinflammatory cytokine IL-17 or a combination of IL-17/IFNγ (double positive) than corresponding c-Met^−^ cells in vitro.

Next, we wondered whether the CD4^+^c-Met^-^ and CD4^+^c-Met^+^ subsets expressed different integrin profiles after activation. We assessed the expression of VLA4 (α4β1), LPAM (α4β7), and LFA-1 (αLβ2), three major integrins expressed by T cells, after anti-CD3/CD28 activation of PBMCs from four HDs. Flow cytometry showed increased expression of the Itgα4 subunit (CD49d) by the CD4^+^c-Met^+^ subset compared to the corresponding c-Met^−^ counterparts. However, no differences were observed in the expression of the other VLA4 subunit, β1 (CD29), the LPAM subunit (β7), or LFA-1 subunits (ItgαL and Itgβ2) (Fig. 1G). Finally, the expression of integrin β3 (CD61) and integrin receptor-mediated CD44, which are known to be involved in T cell migration by association with Itgα4, were not different between the two CD4^+^ T cell subsets (Fig. S3A-B).

We then tested whether the overexpression of Itgα4 on activated CD4^+^c-Met^+^ T cells results in an increased adhesion capacity. We blocked the Itgα4-mediated leukocyte-endothelial cell interaction with a monoclonal antibody, anti-Itgα4 (natalizumab), which is known to inhibit the trafficking of lymphocytes from the blood into the CNS [10]. FACS of CD4^+^c-Met^−^ and CD4^+^c-Met^+^ T cells was performed 72 h after activation, and then the selected cells were seeded on a monolayer of HUVECs (in the presence or absence of TNFα) for 1 h. As shown in Fig. 1H, both CD4^+^c-Met^+^ and CD4^+^c-Met^−^ T cells were able to adhere to TNFα-activated HUVECs. Notably, after anti-Itgα4 blockade, 70% of CD4^+^c-Met^+^ T cells (672 ± 75 to 197 ± 25 cells) but only 35% of CD4^+^c-Met^−^ T cells (535 ± 59 to 337 ± 109 cells) lost their adhesion capacities (Fig. 1H, right panel).

To investigate the effect of the Itgα4 subunit on CD4^+^c-Met^+^ cell trafficking in vitro, we performed a Transwell assay in which a HUVEC monolayer with or without TNFα activation was established in the top chamber and the chemokine CXCL12 was added in the lower chamber. As in the adhesion assay, FACS-sorted CD4^+^c-Met^−^ and CD4^+^c-Met^+^ T cells were seeded in the top chamber and incubated for 6 h after 72 h of activation in the presence or absence of Itgα4 antibody (natalizumab). The transmigration capacity of CD4^+^c-Met^+^ cells was significantly higher than that of CD4^+^c-Met^−^ T cells (Fig. 1I). Again, the blockade of Itgα4 altered the number of transmigrated T cells for both subsets, but the inhibition was higher for CD4^+^c-Met^+^ cells (52% inhibition; from 10244 ± 810 to 4769 ± 993 cells) than for their c-Met^−^ counterparts (27% inhibition; from 6435 ± 236 to 4157 ± 315 cells); Fig. 1I, right panel). These data show that activated CD4^+^c-Met^+^ T cells have a greater ability to adhere and transmigrate through endothelial cells, at least partially due to the overexpression of Itgα4 on their surface.

As our in vitro experiments with fresh human PBMCs may mimic what occurs in an inflammatory environment, we determined whether circulating CD4^+^ T cells express c-Met in patients with inflammatory diseases, such as multiple sclerosis (MS) or cancer (i.e., malignant melanoma, MM). We collected PBMCs from HDs, MS patients, and MM patients and stained for CD4^+^c-Met^+^ T cells. Immunofluorescence of PBMCs subjected to a Cytospin protocol identified CD4^+^c-Met^+^ T cells in MS and MM patients but barely detectable cell counts in HDs (Fig. 1J). We next assessed c-Met expression on circulating CD4^+^ T cells from HDs, MS patients and MM patients by flow cytometry and observed similar results, with an increased CD4^+^c-Met^+^ T cell population in MS patients and an even greater extent in MM patients compared to that in HDs (Fig. 1K). Although the function of these cells in vivo remains to be understood, these data confirm that CD4^+^c‐Met^+^ T cells are readily detectable in human pathological conditions. This observation suggests a novel implication of the HGF/c-Met pathway in the development/maintenance of autoimmune diseases and cancer.

In conclusion, our study uncovered the presence of human c-Met-expressing CD4^+^ T cells upon TCR triggering. Phenotypic and functional analyses of CD4^+^c-Met^+^ T cells revealed an enhanced proinflammatory phenotype skewed toward Th17 and Th17.1 polarization, with increased production of IL-17 either alone or in conjunction with IFNγ (double positive) and higher levels of Itgα4 compared to the levels in CD4^+^c-Met^-^ T cells. Furthermore, anti-Itgα4 treatment directly restrained the adhesion and transmigration capacity of CD4^+^c-Met^+^ T cells in vitro. CD4^+^c-Met^+^ T cells could be detected directly ex vivo from PBMCs from patients with inflammatory conditions, including MS and MM. Our study offers a perspective of previously unsuspected c-Met signaling in activated CD4^+^ T cells, which requires further exploration to better understand the functionality and potential clinical applications of targeting these cells in patients with inflammatory conditions.

## Supplementary information

Methodes and legend Figure S1-3

Table S1

Figure S1

Figure S2

Figure S3

## Data Availability

The datasets used and/or analyzed during the current study are available from the corresponding author upon reasonable request.
