# Supplementary material for: c-Met enforces proinflammatory and migratory features of human activated CD4+ T cells
Source: Cell Mol Immunol. 2021 Jun 28;18(8):2051–3. doi: 10.1038/s41423-021-00721-9 (PMC8322317; doi:10.1038/s41423-021-00721-9)
Supplement: Supplementary file 1 — Methodes and legend Figure S1-3 [file 41423_2021_721_MOESM1_ESM.docx]

**SUPPLEMENTARY INFORMATIONS**

**Methods**

**Standard protocol approvals**

Venous blood was drawn from HDs (3 males and 5 females, mean age 33 ± 12 years), at the Swiss Transfusion Center (Swiss Red Cross) of Geneva under local ethical approval. Volunteers were asked to read an information sheet for blood donation and to complete an online medical questionnaire on the day of donation. After the questionnaire was finalized, a PDF file was generated for printing and signed to give approval for blood donation. MS patient blood (2 males and 1 female, mean age 34 ± 7 years; EC Approval 02724) and malignant melanoma (MM) patient blood (3 males; EC Approval 188/12) was collected upon informed consent. PBMCs were isolated and immediately used or cryopreserved in accordance with the ethical committee of Geneva Hospital (Switzerland).

**Cell lines and condition medium**

Huh7 hepatocytes and HaCaT keratinocytes were cultured in DMEM medium with 4.5 g/L D-glucose, L-glutamine pyruvate supplemented with 10% fetal bovine serum (Sigma), penicillin/streptomycin, and 5% HEPES buffer. Human lymphoma-derived T2 cells were cultured in IMDM medium with GlutaMAX supplemented with 10% fetal bovine serum, penicillin/streptomycin, and 5% HEPES buffer.

**Cell isolation and culture conditions**

PBMCs were obtained by density gradient centrifugation of human peripheral blood over Ficoll–Paque^TM^ PLUS (GE Healthcare, Life Science). Isolated PBMCs were resuspended in complete culture medium (RPMI 1640 medium supplemented with 10% fetal bovine serum, 4 mM L–glutamine (Gibco), 0.1% β-mercaptoethanol, L-asparagine and L-arginine (Sigma Aldrich), 25 mM Hepes buffer, 50 U/ml penicillin, and 50 mg/ml streptomycin; all components purchased from Life Technologies). In selected experiments, PBMCs were activated in the presence of 100 IU/ml recombinant human IL-2 and different activation molecules: 2 μg/ml phytohemagglutinin (PHA, Thermo Fisher), 100 pg/ml lipopolysaccharide (LPS, Sigma Aldrich), or anti-CD3/CD28-coated beads (1:1 ratio, Thermo Fisher) for 72 h at 37°C and 5% CO_2_. To determine the intracellular cytokine content, anti-CD3/CD28-activated PBMCs were incubated with 1 μg/ml ionomycin, 20 ng/ml phorbol 12-myristate 13-acetate (PMA), and 10 μM of the protein transport inhibitor Brefeldin-A (all from Sigma Aldrich) for 4 h according to the manufacturer’s recommendations. For functional experiments, CD4^+^ T cells were isolated using the CD4^+^ T cell negative selection kit (EasySep, StemCell Technologies) according to the manufacturer’s instructions. The purity of isolated cells was always >95% as assessed by flow cytometry using CD4 and CD3 co-staining. For *in vitro* analysis of the effects mediated by anti-Itgα4 (natalizumab), PBMCs from HDs were incubated with 100 μg/ml of anti-Itgα4 during the whole anti-CD3/CD28 activation process (72 h).

**Immunological markers and flow cytometry**

T cells were stained using fluorochrome-conjugated Abs (**Table S1**) in FACS buffer (DPBS free calcium and magnesium, and supplemented with 0.5% bovine serum albumin). Human Fc-block (BD Pharmingen) was used to block unspecific binding by Fc-receptors. The expression of pro-inflammatory cytokines was detected in fixed/permeabilized CD3^+^CD4^+^ T cells using the anti-human IFNγ and anti-human IL-17 Staining Kit (eBioscience). To exclude dead cells, samples were co-stained with DAPI (Invitrogen) or LIVE/dead^TM^ AQUA (Thermo Fisher) and analyzed using a LSRFortessa^TM^ flow cytometer (BD Bioscience) with standard equipment.

**RNA isolation and quantitative real-time PCR**

Total RNA was prepared from MACS-sorted CD4^+^ T cells, followed by separation with FACSAria^TM^ on CD4^+^c-Met^-^ or CD4^+^c-Met^+^ T cells, or T2 cells as a negative control and Huh7 and HaCaT cells as positive controls. Total RNA was extracted using the Qiagen RNeasy Mini Kit and subjected to DNase I (Roche Diagnostics) digestion. Random hexamer primers (Promega) and Superscript II RNase H reverse transcriptase (Invitrogen) were used to generate cDNA. All transcripts were quantified by real-time PCR on a 7500 Real-Time PCR System with Power SYBR Green detection agent (Applied Biosystems). The following primers were used: c-Met (forward: AGCAATGGGGAGTGTAAAGAGG; reverse: CCCAGTCTTGTACTCAGCAAC) and β-actin (forward: CATGTACGTTGCTATCCAGGC; reverse: CTCCTTAATGTCACGCACGAT). PCR was performed using the Taq Platinum polymerase kit (Thermo Fisher). The amplification procedure was 3 min at 94°C followed by 35 cycles of 30 s at 94°C for denaturation, 30 s at 55°C for hybridization, and 1 min at 72°C for elongation. Final elongation was performed for 4 min at 72°C. PCR products were visualized on a 1% agarose gel using Gelgreen (Ozyme) dye under ultraviolet illumination. The expression level of β-actin was used for normalization. For all reactions, each condition was performed in triplicate.

**Western blot analysis**

After 72 h of activation with anti-CD3/CD28-coated beads, CD4^+^ T cells were isolated from PBMCs by FACSAria^TM^ (BD Bioscience) separation. Sorted CD4^+^ T cells, T2, Huh7, and HaCaT cells, were then homogenized using lysis buffer (50 mM Tris–HCl [pH 7.5], 250 mM NaCl, 1% Triton X‐100, 1 mM EDTA, and 1 mM DTT) containing complete protease inhibitors (Roche). Total protein content was determined using the micro BCA^TM^ protein assay kit (Thermo Fisher). Equal amounts (20 μg) of total protein were transferred from each sample to a 15% sodium dodecyl sulfate–polyacrylamide gel and blotted onto an Immobilon‐P polyvinylidene difluoride membrane (Millipore). Expression of c‐Met was detected using properly diluted (1:100) human monoclonal anti‐c‐Met Ab (clone EP1454Y, Abcam, AB_765024), followed by a peroxidase‐conjugated secondary Ab to rabbit IgG (Invitrogen), and then visualized using chemiluminescence (Supersignal, Pierce). The blot was also probed with mouse monoclonal anti‐β‐actin (clone 15G5A11/E2) as a loading control (Sigma Aldrich, AB_476744).

**Immunofluorescence and immunohistochemistry**

Cytospin PBMCs from HDs (activated with anti-CD3/CD28 or not), MS patients, and MM patients were fixed with 4% PFA. Slides were blocked for 30 min in PBS with 1% bovine serum albumin. Abs against human c-Met (EP1454Y, Abcam, AB_765024), CD4 (4B12, Bio-Rad, MCA6002), or CD44 (IM7, Biolegend, AB_312952) were applied at 1:100 dilution. Sections were incubated overnight with Abs, followed by several washes with PBS. Secondary anti-rabbit and anti-mouse (Invitrogen) Abs were used at 1:500 dilution. Slides were mounted with ProLong Gold anti-fade reagent with DAPI (Life Technologies) and kept at 4°C. The slides were imaged in the bioimaging facility of the University of Geneva using a Leica SP5 Axiocam system (Leica MicroImaging). At least three acquisitions were performed for the same donor, and the most representative images were selected.

**Cell adhesion assay**

Human umbilical vein endothelial cells (HUVECs) were cultured to confluence on 96-well plates, and then treated with or without 10 ng/ml TNFα. PBMCs from HDs were treated with or without anti-Itgα4 (100 μg/ml) for the whole anti-CD3/CD28 activation period (72 h). FACS-sorted CD4^+^c-Met^-^ and CD4^+^c-Met^+^ activated T cells were then labeled with either 5 μM CellTrace™ carboxyfluorescein diacetate succinimidyl ester (CFSE, Thermo Fisher) or 5 μM CellTrace™ Far Red (Thermo Fisher) according to the manufacturer’s instructions. Both stained cells (50/50 ratio) were then seeded on a confluent monolayer of HUVECs for 1 h at 37°C. After incubation, the unbound CD4^+^ T cells were rinsed away three times and pictures of five fields were taken of each well using a ZOE™ Fluorescent Cell (Bio-Rad). The number of attached cells was counted using CellProfiler^TM^ software.

**Transwell assay**

Transwell migration assays were performed to assess CD4^+^c-Met^-^ and CD4^+^c-Met^+^ T cell transmigration through a monolayer of TNFα-activated endothelial cells in the presence of CXCL12/SDF-1α (100 ng/ml) chemoattractant in the bottom chamber. HUVECs were coated on the transwell chamber (24-well insert, diameter: 6.5 mm, pore size: 5 µm; Corning, Vitaris AG). PBMCs from HDs were treated with or without anti-Itgα4 (100 μg/ml) for the whole anti-CD3/CD28 activation period (72 h). FACS-sorted CD4^+^c-Met^-^ or CD4^+^c-Met^+^ activated T cells (3x10^4^) were then seeded into the top chamber and incubated for 6 h at 37°C to allow the cells to attach and transmigrate. The cells in the lower chamber were harvested and the relative number of transmigrated cells determined by flow cytometry using fluorescent counting beads (Thermo Fisher) to normalize the number of transmigrated cells.

**Statistical analysis**

GraphPad Prism 8 software was used to perform the statistical analyses. Unpaired two-tailed Student’s t-tests were used when comparing two groups. Intergroup comparisons were conducted by two-way analysis of variance (ANOVA) followed by Tukey’s post hoc test for comparisons of multiple groups. P < 0.05 was considered statistically significant. If not mentioned, differences are not statistically significant.

**Figure S1. CD4^+^ T cells express the HGF receptor c-Met at a very weak level in the peripheral blood of healthy donors (HDs)**

**(A)** Flow cytometry gating strategy for the identification of c-Met-expressing CD4^+^ T cells. Live lymphocytes were first selected for their morphology using FSC/SSC parameters, followed by the exclusion of dead cells (DAPI) and doublets. CD3^+^CD4^+^ T lymphocytes were subsequently selected and Th0 cells (CD45RA^+^CCR7^+^) separated from differentiated CD4^+^ T cells. CXCR3, CCR6, and CRTH2 markers were used to identify different subpopulations of T helper cells: Th1 (CXCR3^+^CCR6^-^), Th2 (CXCR3^-^CCR6^-^CRTH2^+^), Th17 (CXCR3^-^CCR6^+^), and Th17.1 (CXCR3^+^CCR6^+^).

**(B)** RNA-Seq data showing relative log c-Met expression post-normalization in sorted Th1, Th2, Th17, and Th17.1 cells from HD PBMCs. Normalized read counts across all samples were used for subsequent analyses. Data are presented as mean ± SEM for *n* = 3 HDs.

**(C)** Representative flow cytometry histograms of c-Met expression in the T helper cell subpopulations described in (A). Grey-filled histograms represent fluorescence minus one (FMO) to determine background fluorescence.

**(D)** Quantification of c-Met geometric mean fluorescence intensity (Gmean) for each subpopulation showed in (C). Data are presented as mean ± SEM for *n* = 8 HDs.

**Figure S2. Activated CD4^+^ T cells express c-Met and their distribution into Th population is different to CD4^+^c-Met^-^ T cells**

**(A)** Relative expression of c‐Met mRNA in FACS‐sorted non-activated and anti-CD3/CD28-activated CD4^+^ T cells was assessed by real-time PCR. T2, Huh7, and HaCaT cells were used as negative and positive controls, respectively. Values were normalized using β-actin. Data are presented as mean ± SEM for n = 5 HDs; ****P ≤ 0.0001 by unpaired two‐tailed Student's t‐test.

**(B)** Western blot of the expression of c-Met in 1) T2 cells (negative control), 2) Huh7 cells (positive control), 3) HaCaT cells (positive control), 4) fresh FACS-sorted CD4^+^ T cells from HD, and 5) FACS-sorted CD4^+^ T cells after anti-CD3/CD28 activation. β-actin was the housekeeping gene used as a protein control. Data are representative of *n* = 5 HDs.

**(C)** Pie charts depicting the mean distribution of Th1 (CXCR3^+^CCR6^-^), Th2 (CXCR3^-^CCR6^-^CRTH2^+^), Th17 (CXCR3^-^CCR6^+^), Th17.1 (CXCR3^+^CCR6^+^), and the other CD4^+^ T subsets within the c-Met^-^ (left panel) and c-Met^+^ (right panel) populations after anti-CD3/CD28 activation.

**Figure S3. Activated CD4^+^c-Met^+^ and CD4^+^c-Met^-^ T cells express similar levels of Itgβ3 and CD44**

**(A)** Representative flow cytometry histograms and quantification of Itgβ3/CD61 expression in CD4^+^c-Met^-^ and c-Met^+^ T cells activated with anti-CD3/CD28-coated beads. Data are presented as mean ± SEM for n = 4 HDs.

**(B)** Representative flow cytometry plots and quantification of integrin receptor-mediated CD44 expression in CD4^+^c-Met^-^ and c-Met^+^ T cells activated with anti-CD3/CD28-coated beads. Data are presented as mean ± SEM for n = 4 HDs.
