# Supplementary material for: c-Met enforces proinflammatory and migratory features of human activated CD4+ T cells
Source: Cell Mol Immunol. 2021 Jun 28;18(8):2051–3. doi: 10.1038/s41423-021-00721-9 (PMC8322317; doi:10.1038/s41423-021-00721-9)
Supplement: Supplementary file 2 — Table S1 [file 41423_2021_721_MOESM2_ESM.docx]

**Table S1**: Antibodies for flow cytometry

| **Specificity** | **Clone** | **Supplier** | **RRID number** |
| --- | --- | --- | --- |
| c-Met | 95106 | R&D Systems | AB_1026292 |
| CRTH2 | BM16 | Biolegend | AB_10900060 |
| CD3 | UCHT1 | Biolegend | AB_893299 |
| CD4 | OKT4 | Biolegend | AB_571959 |
| CD45RA | HI100 | Biolegend | AB_314416 |
| CCR7 | G043H7 | Biolegend | AB_10915137 |
| CCR6 | G034E3 | Biolegend | AB_2562235 |
| CXCR3 | G025H7 | Biolegend | AB_2563533 |
| IL-17 | ebio64DEC17 | eBioscience | AB_10596502 |
| IFNγ | 4S.B3 | eBioscience | AB_1311247 |
| CD49d/Itgα4 | 9F10 | Biolegend | AB_2687198 |
| CD29/Itgβ1 | TS2/16 | Biolegend | AB_314324 |
| CD11a/ItgαL | HI111 | Biolegend | AB_314144 |
| CD18/Itgβ2 | TS1/18 | Biolegend | AB_314226 |
| CD61/Itgβ3 | 23C6 | Biolegend | AB_2810448 |
| Itgβ7 | FIB504 | Biolegend | AB_571971 |
| CD44 | IM7 | Biolegend | AB_493687 |
