# Supplementary figures and images for: c-Met enforces proinflammatory and migratory features of human activated CD4+ T cells
Source: Cell Mol Immunol. 2021 Jun 28;18(8):2051–3. doi: 10.1038/s41423-021-00721-9 (PMC8322317; doi:10.1038/s41423-021-00721-9)

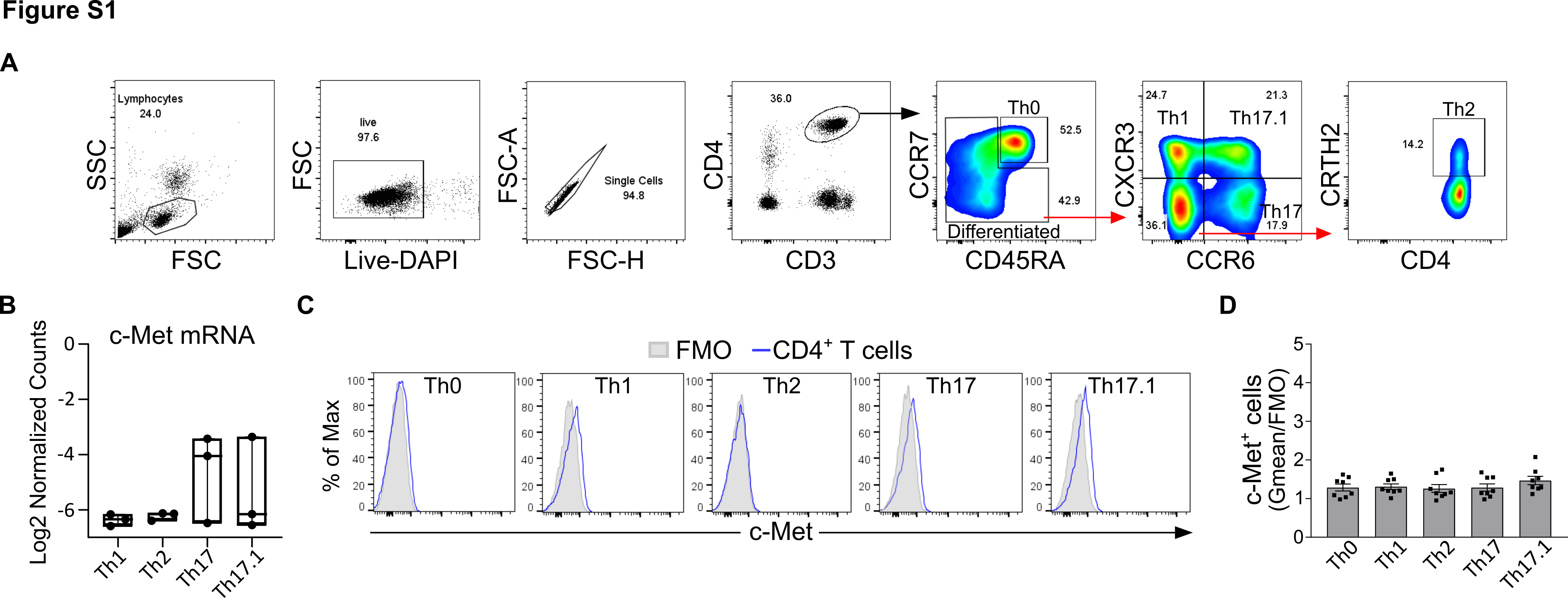

Supplement: Supplementary file 3 — Figure S1 [file 41423_2021_721_MOESM3_ESM.png]

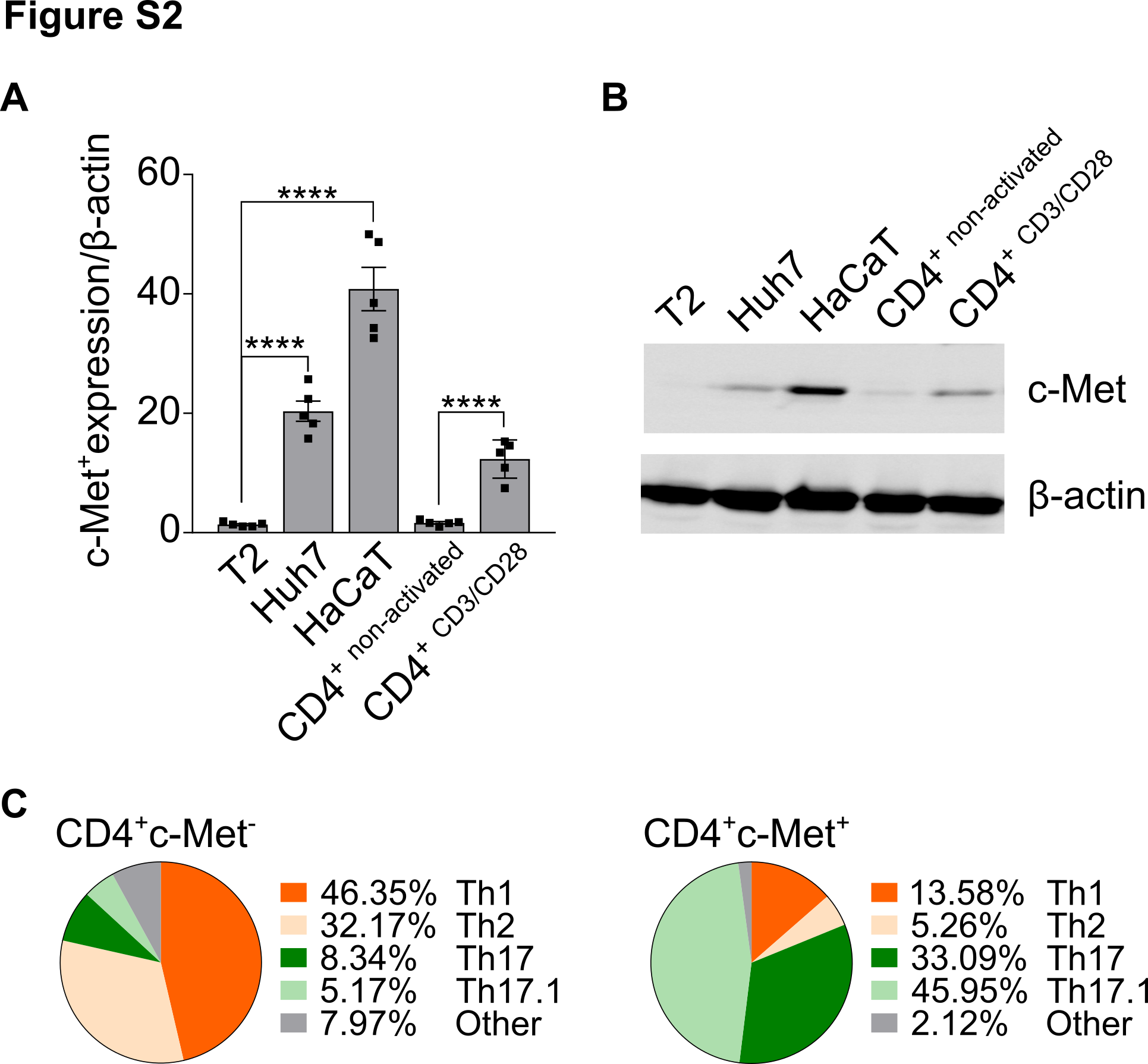

Supplement: Supplementary file 4 — Figure S2 [file 41423_2021_721_MOESM4_ESM.png]

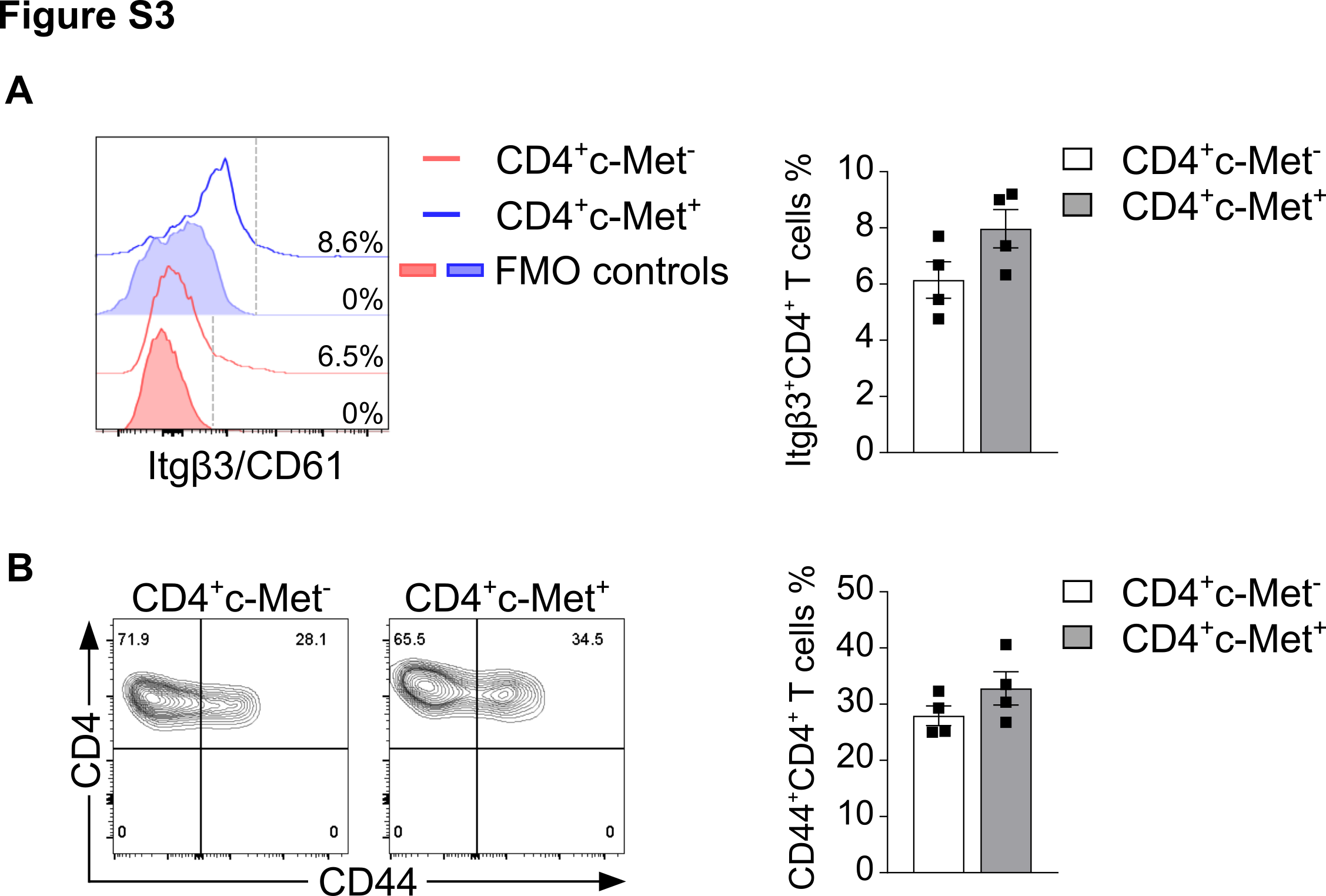

Supplement: Supplementary file 5 — Figure S3 [file 41423_2021_721_MOESM5_ESM.png]
